# Supplementary material for: Lignin-Based Nanocarrier for Simultaneous Delivery of 131I and SN-38 in the Combined Treatment of Solid Tumors by a Nanobrachytherapy Approach
Source: Pharmaceuticals (Basel). 2025 Jan 27;18(2):177. doi: 10.3390/ph18020177 (PMC11859004; doi:10.3390/ph18020177)
Supplement: Supplementary file 1 [file pharmaceuticals-18-00177-s001.zip › pharmaceuticals-3416242-supplementary.pdf]

Supplementary Materials

Lignin-Based Nanocarrier for Simultaneous Delivery of ^{131}I and SN-38 in the Combined Treatment of Solid Tumors by a Nanobrachytherapy Approach

Aleksandar Vukadinović [1], Miloš Ognjanović [1], Milica Mijović [2], Bryce Warren [3], Slavica Erić [4] and Željko Prijović [1,*]

[1] "Vinča" Institute of Nuclear Sciences-National Institute of the Republic of Serbia, University of Belgrade, 11351 Belgrade, Serbia
[2] Institute of Pathology, Faculty of Medicine, University in Priština-Kosovska Mitrovica, 38220 Kosovska Mitrovica, Serbia
[3] Natural State Science LLC, 415 N. McKinley Street, Little Rock, AR 72205, USA
[4] Faculty of Pharmacy, University of Belgrade, 11221 Belgrade, Serbia
* Correspondence: zprijovic@vin.bg.ac.rs

1. Preparation of on lignin nanoparticles and the loading with SN-38

1.1 The synthesis of the nanoparticles

To optimize the synthesis and the loading parameters and to determine amount of SN-38 in lignin-SN-38 nanoparticles, solution of lignin alone (10 mg/ml in DMSO (good solvent)) was mixed with solution of SN-38 (4 mg/ml, 2 mg/ml, 1 mg/ml and 0.5 mg/ml in DMSO) in ratio 1:1(v/v), giving final mixtures containing 5 mg/ml Lignin with, 2 mg/ml, 1 mg/ml, 0.5 mg/ml and 0.25 mg/ml SN-38. One ml (1ml) of the solution containing the lignin alone or the mixtures was placed in DMSO pre-wet dialysis membrane of cut-off 14 kDa and dialyzed against pure water with pH adjusted by HCl to 5.2-5.3 during 2-6 h. After that, the dialysis bags are placed into water pH 6.5 and kept 20 h more. The final dialysates were either used to determine the size by dynamic light scattering or transferred to a glass vial and freeze-dried 24 h. Obtained nanomaterial was in form of fluffy light-brown powder, which is used for FTIR spectra. For the radiolabelling and the therapy, the material is re-constituted in PBS as described.

The parameters chosen to produce the material for in vivo application were as follows: ratio of SN-38 to lignin 1:5 (m/m), 4 h dialysis at pH = 5.2-5.3, 20 h dialysis in water pH = 6.5 with one exchange of the buffer. To estimate the reproducibility of the process, the protocol was repeated in triplicate. The results are presented below. The smallest lignin and lignin-SN-38 nanoparticles were chosen for the *in vivo* experiments.

Lignins: Lignin + SN-38

No	Size (nm)	SD	PDI*	No.	Size (nm)	SD	PDI*
Lignin 1	170.20	75.96	0.20	Lig+SN-38 1	107.00	44.92	0.18
Lignin 2	86.84	32.24	0.14	Lig+SN-38 2	119.60	63.44	0.28
Lignin 3	106.20	54.96	0.27	Lig +SN-38 3	217.90	91.71	0.18
AV	121.08	54.38	0.20	AV	143.17	66.69	0.21

*PDI is calculated as $(SD)^2/(AV)^2$, where AV are averages and SD is the standard deviation.

Table S1: Characterization of the synthesized nanomaterial by Dynamic Light Scattering

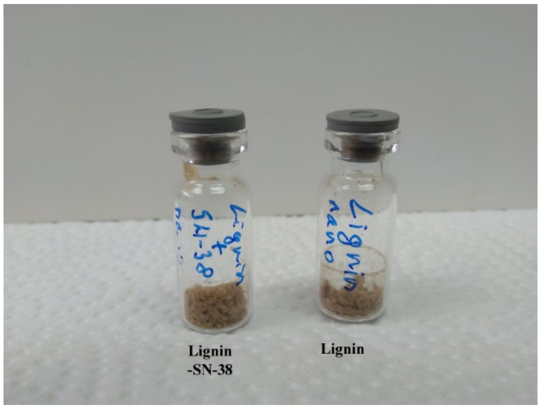

Figure S1: Lignin and lignin-SN-38 nanomaterial

1.2.FTIR spectra of the nanomaterial

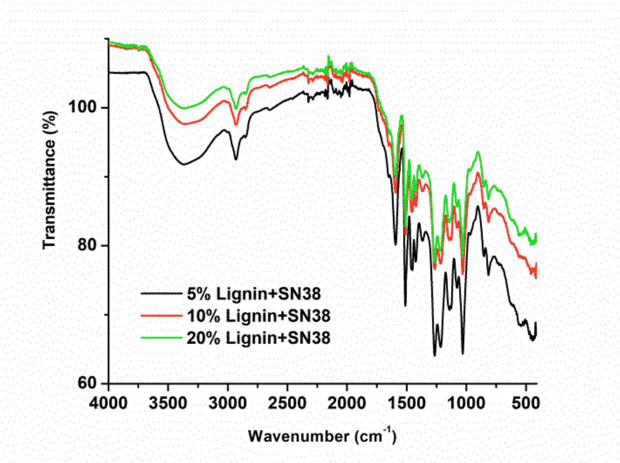

Figure S2: FTIR spectra of nanoparticles with different SN-38 /Lignin ratios

1.3 The analysis of the SN-38 contents of the nanoparticles by SPE-HPLC

To quantitate the content, a sample of the powder was accurately weighted at 4 decimal scale and dissolved into DMSO at 1 mg/ml concentration. The solution was further serially

diluted 2x10 fold in DMSO then again 2x10 fold in the SPE mobile phase. The 10^4 dilution injected into SPE-HPLC equipped with 50 µl loop.

For standard curve, 10 mg/ml SN-38 in DMSO was serially diluted 2x10 fold by DMSO, then 2x10 fold by mobile phase, to get 100 ng/ml. Then, from this stock, additional dilution was made to generate 2, 4, 6, 8 and 10 ng/ml by taking 20, 40, 60, 80 and 100 µl of the stock and filling up to 1 ml. The samples were injected in triplicates into HPLC as 50 µl volume. The fluorescent signal was detected at ex-375 emm 540nm.

The HPLC method: adapted from our earlier method for CPT-11 and its metabolites in biological material [1].

The HPLC system is assembled from two Waters 626 pumps and controllers, a µBondapack SPE and a µBondapack analytical column, a Gilson Valvemate 6 port valve, the Waters UV/Vis spectrophotometer detector and the JASCO FP-980 fluorescence detector, all controlled by a N2000 HPLC data logging system.

The method achieves the separation and concentration of SN-38 from lignin and other impurities on an SPE column under low acetonitrile concentration, followed by the transfer of the trapped SN-38 to the analytical column and its analysis under high acetonitrile conditions in high salt and low pH mobile phase.

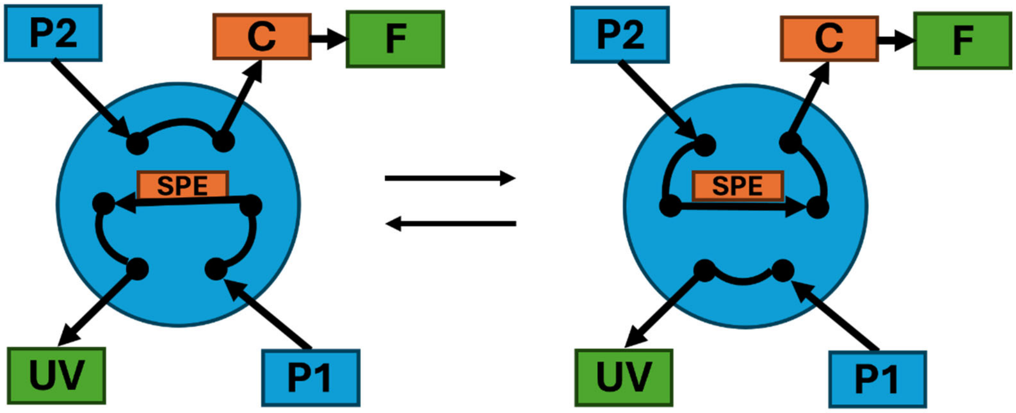

Figure S3: The 6-port valve setup for SPE HPLC:

P1, P2-Pumps, SPE-Solid Phase Extraction column, C-analytical column, UV, F-detectors

The dry powder containing free or lignin-bound SN-38 was dissolved into DMSO, followed by an appropriate serial dilution (commonly four serial tenfold dilutions) in the low acetonitrile mobile phase (5% ACN in 0.025 M K-phosphate pH-2.9) and injected into the HPLC system equipped with 100 µl loop running the mobile phase at 1ml/min by pump 1 and the 6-port

valve at position 1, directing the liquids through UV/Vis detector set up to 280 nm. After 2.5 min (the time well enough to separate and wash away the lignin and impurities), the 6-port valve was switched to position 2, which re-directs flow of pump 2, running the analytical mobile phase (26% ACN in 0.1 M K-phosphate pH=2.9) in the opposite direction through the SPE column, dissolving the analytes and carrying them further to the analytical column for separation, as detected on the fluorescence detector set to excitation of 375 nm and emission at 540 nm. The spectra were acquired, integrated, and analyzed by an N2000 HPLC data logging system.

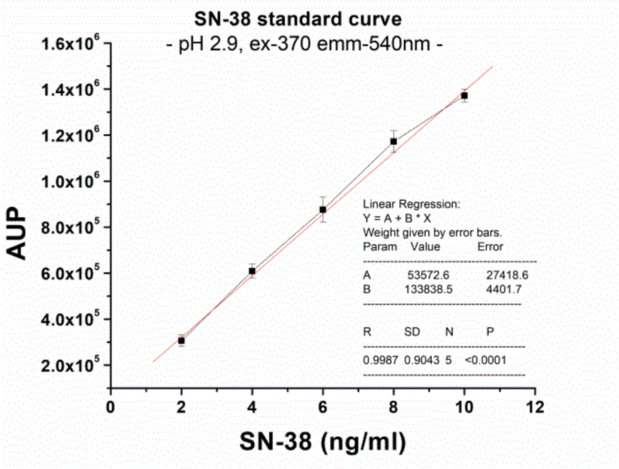

Figure S4: The HPLC standard curve of SN-38

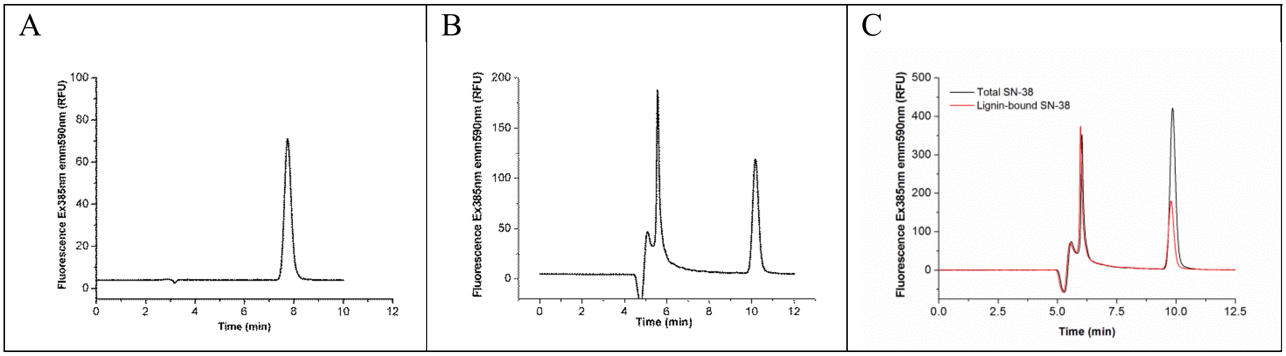

Figure S5. Examples of chromatograms of SN-38: injected directly (A), injected in SPE (B) and overlapped lignin extracts in SPE (C).

2. The in vivo distribution

Figure S6: An overlapped *in vivo* image of a 4T1 tumor xenograft-bearing male mouse injected with 250 µg of ^{131}I-SN-38@Lignin containing 50 µg of SN-38 and 1.75 MBq of ^{131}I in 50 µl of saline, 1h after the injection. The red color is the luciferase-generated light from the tumor cells, green color is the radiation, and the yellow color is the co-localization of the two.

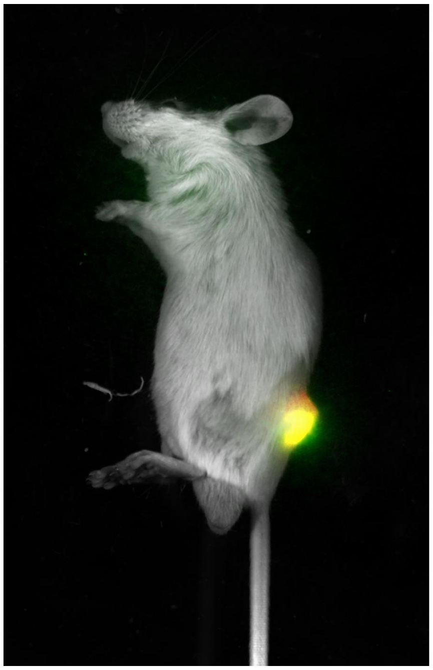

3. The therapeutic effect of the nanomaterial

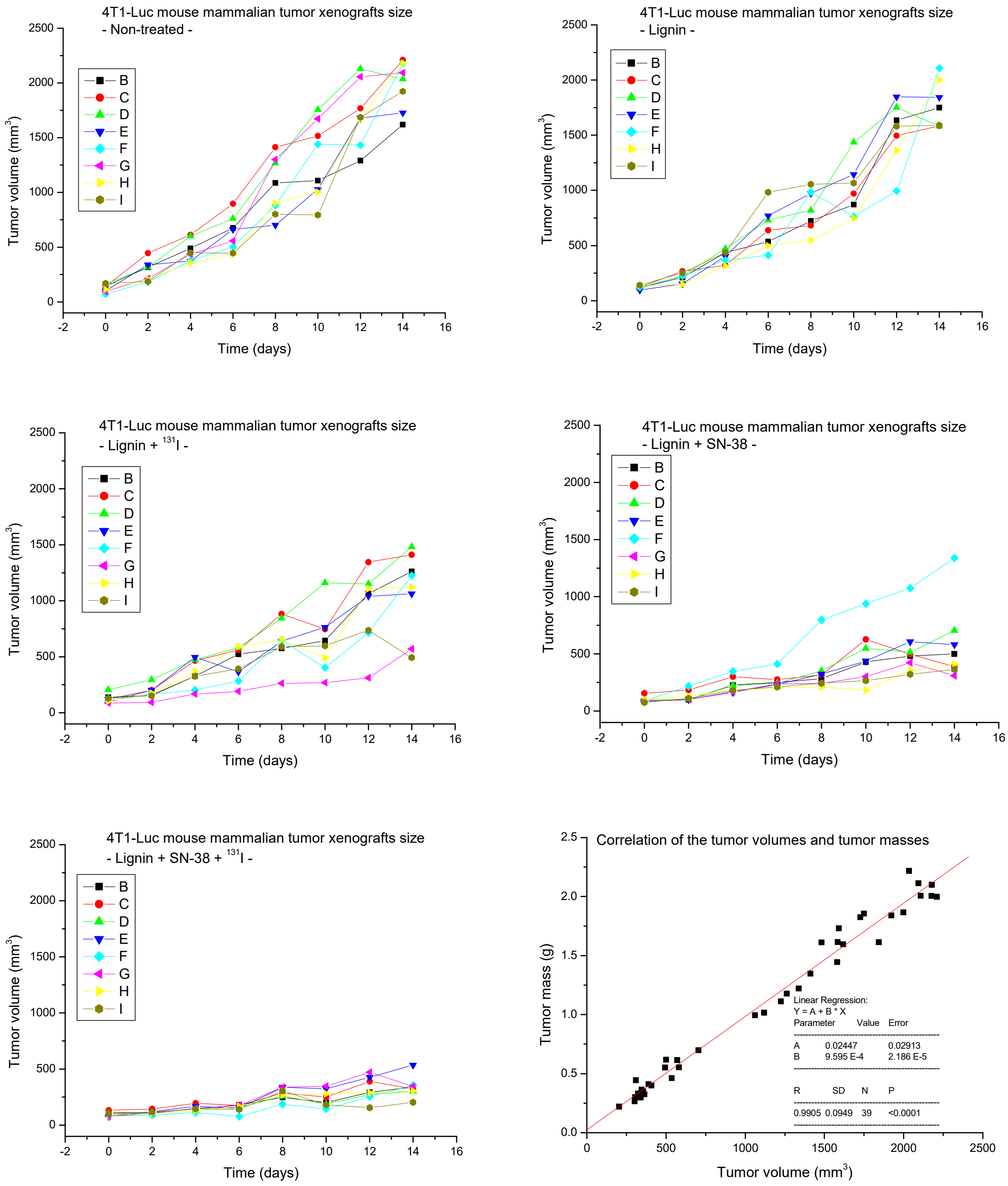

Figure S7: The individual tumors growth and the correlation of the measured volume and real tumor mass at the end of experiment at day 14th.

4. References:

(1) Prijovich, Z. M.; Burnouf, P.-A.; Roffler, S. R. Versatile online SPE–HPLC method for the analysis of Irinotecan and its clinically relevant metabolites in biomaterials. *Journal of Separation Science* **2014,** *37* (4), 360-367, DOI: https://doi.org/10.1002/jssc.201301191.
